# Correction: Structure, magnetism and heating ability of pyrrole-functionalized magnetic biochar (PFMB) for magnetic hyperthermia

**DOI:** 10.1039/d5ra90099a

**Published:** 2025-09-01

**Authors:** O. M. Lemine, M. Bououdina, Turki Altoub, M. Alshammari, Kadi Y. Museery, Ali Z. Alanzi, Latifa Latrous

**Affiliations:** a Department of Physics, College of Science, Imam Mohammad Ibn Saud Islamic University (IMSIU) Riyadh 11623 Saudi Arabia mamamin@imamu.edu.sa; b Department of Mathematics and Sciences, College of Humanities and Sciences, Prince Sultan University 11586 Riyadh Saudi Arabia; c Microelectronics and Semiconductors Institute, King Abdulaziz City for Science and Technology (KACST) Riyadh Saudi Arabia; d Laboratoire de Chimie Minérale Appliquée (LR19ES02), Faculté Des Sciences de Tunis, Université de Tunis El Manar Campus Universitaire El Manar I 2092 Tunis Tunisia

## Abstract

Correction for “Structure, magnetism and heating ability of pyrrole-functionalized magnetic biochar (PFMB) for magnetic hyperthermia” by O. M. Lemine *et al.*, *RSC Adv.*, 2025, **15**, 28145–28154, https://doi.org/10.1039/D5RA04120A.

The authors regret that affiliation *a* was shown incorrectly in the original manuscript. The corrected list of affiliations is as shown herein.

The Royal Society of Chemistry apologises for these errors and any consequent inconvenience to authors and readers.

